# Musculoskeletal Strength, Balance Performance, and Self-Efficacy in Elderly Ving Tsun Chinese Martial Art Practitioners: Implications for Fall Prevention

**DOI:** 10.1155/2014/402314

**Published:** 2014-10-29

**Authors:** Shirley S. M. Fong, Shamay S. M. Ng, Karen P. Y. Liu, Marco Y. C. Pang, H. W. Lee, Joanne W. Y. Chung, Priscillia L. Lam, X. Guo

**Affiliations:** ^1^Institute of Human Performance, The University of Hong Kong, Pokfulam, Hong Kong; ^2^Department of Rehabilitation Sciences, The Hong Kong Polytechnic University, Hung Hom, Hong Kong; ^3^School of Science and Health, University of Western Sydney, Penrith, NSW, Australia; ^4^Department of Health and Physical Education, Hong Kong Institute of Education, Tai Po, Hong Kong; ^5^Physiotherapy Department, David Trench Rehabilitation Centre, Hong Kong

## Abstract

*Objectives.* To (1) compare the bone strength, lower limb muscular strength, functional balance performance, and balance self-efficacy between Ving Tsun (VT) martial art practitioners and nonpractitioners and (2) identify the associations between lower limb muscular strength, functional balance performance, and balance self-efficacy among the VT-trained participants. *Methods.* Thirty-five VT practitioners (mean age ± SD = 62.7 ± 13.3 years) and 49 nonpractitioners (mean age ± SD = 65.9 ± 10.5 years) participated in the study. The bone strength of the distal radius, lower limb muscular strength, functional balance performance, and balance self-efficacy were assessed using an ultrasound bone sonometer, the five times sit-to-stand test (FTSTS), the Berg balance scale (BBS), and the Chinese version of the activities-specific balance confidence scale, respectively. A multivariate analysis of covariance was performed to compare all the outcome variables between the two groups. *Results.* Elderly VT practitioners had higher radial bone strength on the dominant side (*P* < 0.05), greater lower limb muscular strength (*P* = 0.001), better functional balance performance (*P* = 0.003), and greater balance confidence (*P* < 0.001) than the nonpractitioners. Additionally, only the FTSTS time revealed a significant association with the BBS score (*r* = −0.575,  *P* = 0.013). *Conclusions.* VT may be a suitable health-maintenance exercise for the elderly. Our findings may inspire the development of VT fall-prevention exercises for the community-dwelling healthy elderly.

## 1. Introduction

Falls are common among the elderly and may compromise senior people's physical and psychological health [1]. For example, seniors may suffer from fractured distal radius resulting from falls on outstretched hand [2]. In addition, elderly people who have experienced falls may have greater fear of falling and less confidence in their balance. As a result, they may be less active, which can negatively affect their lower limb muscular strength [3, 4]. Improving musculoskeletal health and balance ability through exercise could reduce falls and perhaps fall-related fractures [2, 5] and also improve balance confidence in the elderly [6].

Ving Tsun (VT) is a traditional Chinese martial art that has the potential to be developed into a new form of health-maintenance exercise to prevent bone loss and the deterioration of balance ability in the elderly [7]. VT involves progressive training with a lot of relatively high-impact osteogenic activities (e.g., wooden dummy training) and functional balance tasks (e.g., sticking-hand exercises) [8]. However, all of these potential physically beneficial effects are underexamined. To the best of our knowledge, no study has investigated VT's psychological benefits (e.g., balance confidence). The major objective of this study was, thus, to compare the bone strength, lower limb muscular strength, functional balance performance, and balance self-efficacy between VT practitioners and nonpractitioners. We also aimed to identify the associations between lower limb muscular strength, functional balance performance, and balance self-efficacy among the VT-trained participants. Our findings shed light on the potential use of VT exercises to avoid falls and the associated fractures in the elderly population.

## 2. Methods

### 2.1. Study Design and Participants

This was a cross-sectional exploratory study. Community-dwelling elderly people who engaged in VT martial arts training (*n* = 35) were recruited from five local VT martial arts associations by convenience sampling. Forty-nine healthy volunteers were recruited from an elderly center in the community to form a comparison group. The inclusion criteria were the following: (1) having regular VT training at least two hours per week for three consecutive months (for the VT group participants only); (2) being 40 years old or above; (3) being able to ambulate independently without the use of walking aids; and (4) being able to communicate and follow verbal commands. The exclusion criteria were the following: (1) having unstable medical conditions such as cardiovascular problems; (2) having a history of significant cognitive, orthopedic, neurologic, visual, vestibular, or cardiopulmonary disorders that may affect their assessment performance; or (3) having experience in martial arts apart from VT (for the VT group participants). This study was approved by the University Ethics Committee and conducted following the principles of the Declaration of Helsinki for human experiments. Written informed consent was obtained from each participant before data collection.

### 2.2. Measurements 

#### 2.2.1. Demographics

Data collection was performed by a physiotherapist and trained assistants in the VT martial art schools and elderly centre. All participants had an interview first, in which relevant information including age, sex, VT experience, and fall history was obtained. The body mass index (BMI) was calculated after body height and weight were measured by a mechanical beam scale (Detecto Physician's Scale, Detecto, MO) or an electronic weight scale. Each of the participants then underwent the following physical assessments in random order.

#### 2.2.2. Bone Strength of Distal Radius

A Sunlight MiniOmni Ultrasound Bone Sonometer (Sunlight, BeamMed Ltd., Israel) was used to assess the bone strength of distal radius of the dominant arm. This system was found to be reliable with an intraoperator precision of 0.36% measured at the distal radius and precise in vivo (0.4%–0.8%) [9–11]. The measurement procedures were presented in Fong et al. [7]. To summarize, each participant was seated with the tested forearm supported on a table. The assessor placed the handheld probe (with the ultrasound transducers inside) at the distal third of the radius and then rotated it slowly around the radial bone without lifting it up from the skin surface. This measurement procedure was repeated three times or more until the speed of ultrasound (SOS) (in ms^−1^) was calculated by the inbuilt computer program. The SOS value represents the velocity of the ultrasound wave traveling through a few centimeters of the tested radius parallel to its axis within the outer 2 to 6 mm [12]. This SOS value was then converted into a *T*-score and a *Z*-score using the same computer program. The SOS *T*-score and SOS *Z*-score refer to the units of standard deviations (SD) relative to the population reference values of ethnicity-matched healthy young adults and ethnicity-, age-, and sex-matched populations, respectively. Both scores reflect the bone's fragility and are associated with bone strength [13]. The SOS *T*-score threshold for the diagnosis of osteoporosis at the distal radius is −2.6 while a value between −2.6 and −1.4 suggests osteopenia [9]. Both the SOS *T*-score and the SOS *Z*-score were used for analysis in this study.

#### 2.2.3. Lower Limb Muscular Strength

For the measurement of lower limb muscular strength, the five times sit-to-stand (FTSTS) test was used. This functional test is widely used to measure lower limb muscular strength and has moderate intrarater reliability (ICC = 0.64) [14] and excellent interrater reliability (ICC = 1.0) [15] in healthy elderly. Detailed assessment procedures were described by Ng et al. [16]. In brief, the participants started in a seated position. They were instructed to stand up and sit down five times as quickly as possible and the whole process was timed. Each participant performed two trials and the average time taken was used for analysis [16].

#### 2.2.4. Functional Balance Performance

The Berg balance scale (BBS), which has been found to be reliable (interrater reliability ICC = 0.98; intrarater reliability ICC = 0.71–0.99) and valid in older adults, was used to determine functional balance [17, 18]. It is a 14-item test with a 5-point ordinal scale (0–4) per item, yielding a maximum total score of 56. A score of zero for an item indicates an inability, or that maximal assistance is required, to complete the task or perform tasks safely, while a score of four indicates that the task can be performed both independently and safely. A BBS total score (i.e., the sum of all item scores) of <45 is predictive of multiple falls [19]. The total score was calculated and used for analysis. The higher the total score, the better the balance ability.

#### 2.2.5. Balance Self-Efficacy

The balance self-efficacy of the participants was evaluated with the validated activities-specific balance confidence (ABC) scale, Chinese version. It has high internal consistency (Cronbach's alpha = 0.97), excellent test-retest reliability (ICC = 0.99), and good interrater reliability (ICC = 0.85) [20]. Participants were asked to rate their self-perceived balance confidence level on an 11-point scale ranging from 0 (no confidence at all) to 100 (completely confident) for completing 16 functional activities without losing balance or becoming unsteady (e.g., walk in a crowded mall). The mean of the total item score (i.e., mean ABC score), ranging from 0 to 100, was used for analysis. Scores close to 100 indicate higher levels of balance self-efficacy [20].

### 2.3. Statistical Analysis

Differences in the characteristics of the VT group participants and the control participants were compared with independent *t*-tests (for continuous data) and chi-square tests (for nominal data). In addition, the Kolmogorov-Smirnov test was used to check the normality of data. To avoid an increase in type I error due to multiple comparisons, multivariate analysis of covariance (MANCOVA) was performed for the (1) bone strength indexes and (2) muscular strength and balance variables. Any significant between-group differences in participant characteristics were treated as covariates in the MANCOVA analyses. Partial eta-squared values (measures of effect size for MANCOVA) were also presented. By convention, partial eta-squared values of 0.01, 0.06, and 0.14 are regarded as small, medium, and large effect sizes, respectively [21]. Moreover, to determine the bivariate correlations among the muscular strength and balance outcomes in the VT-trained participants, Pearson's *r* was used. All statistical analyses were performed using the IBM Statistical Package for Social Sciences 20.0 software (IBM, Armonk, NY, USA). A significance level of 5% was set for all the statistical tests (two-tailed).

## 3. Results

Thirty-five VT-trained participants (VT group) and 49 control participants (control group) fulfilled the eligibility criteria and completed the assessments. The demographic characteristics of the participants are presented in Table 1. The proportion of male participants was significantly higher in the VT group than the control group (*P* = 0.001). The VT group participants were also significantly taller than the control participants (*P* = 0.004) (Table 1). Therefore, sex and body height were treated as covariates in the subsequent statistical analyses.

MANCOVA analysis revealed an overall significant difference in bone strength indexes (Hotelling's Trace = 3.976, *P* = 0.023) and muscular strength and balance outcomes (Hotelling's Trace = 10.196, *P* < 0.001) between the two groups. When each individual outcome was considered, the between-group difference remained significant for all bone strength indexes (*P* < 0.05), muscular strength outcome (*P* = 0.001), and balance outcomes (*P* < 0.01). The SOS *T*-score and SOS *Z*-score, indicating bone strength, were significantly higher in the VT group than the control group by 48.1% (*P* = 0.035) and 77.8% (*P* = 0.009), respectively. In addition, the VT group required 40.3% less time to complete the FTSTS test than the control group (*P* = 0.001), indicating greater lower limb muscular strength in the VT group. The BBS score and ABC score were also significantly higher in the VT group than the control group by 18.9% (*P* = 0.003) and 25.0% (*P* < 0.001), respectively (Table 2). Moreover, partial eta-squared values ranged from 0.055 to 0.384 for all variables of interest, indicating medium to large effect sizes (Table 2).

Correlation analyses were performed for the VT group only. Only the FTSTS time showed a significant association with the BBS score (*r* = −0.575, *P* = 0.013). No significant correlations were found between the FTSTS time and ABC score (*r* = −0.123, *P* = 0.627) and BBS score and ABC score (*r* = 0.109, *P* = 0.665).

## 4. Discussion

### 4.1. Bone Strength

VT-trained older adults had significantly higher bone strength than the control group seniors, after adjusting for sex and body height. This finding is anticipated and actually in agreement with one of our previous studies showing that radial bone strength was higher in long-term VT practitioners (males exclusively in their fifties) than the age- and sex-matched controls [7]. VT training includes many striking movements using the forearms, for example, sandbag workouts, sticking-hand exercises, and wooden dummy training [8, 22], and these movements repetitively load the forearm bones (including the radius) with high impact forces, resulting in remodeling and strengthening of these bones to withstand the external loads or stress [23]. This phenomenon is best explained by Wolff's law, which states that the internal structure of a bone is adapted to mechanical demands such that the trabecular orientation coincides with the stress trajectories [24].

We are specifically concerned with the bone strength of the distal radius because Colles' fractures or wrist fractures are the most common upper extremity fracture resulting from falls on the outstretched hand in older adults [25]. If VT training can improve radial bone strength, it could serve as Colles' fracture-prevention exercise and could be incorporated into the traditional fall-prevention programs. Certainly, further randomized controlled trials should be carried out to prove its effectiveness in strengthening the forearm bones.

### 4.2. Lower Limb Muscular Strength

This is the first study to report that VT practitioners had greater lower limb muscular strength, as reflected by their shorter completion time in the FTSTS test, than the nonpractitioners. VT-trained participants required only 8.3 s to complete the FTSTS test while the control participants required 13.9 s and the age-matched norm is 12.1 s [26]. The present finding is basically in line with previous studies showing that hard-style martial arts practitioners and karate athletes had greater knee muscular strength than control groups [27, 28]. Although VT is relatively softer (less focused on impact) than karate and its training moves away from forceful kicking techniques, practitioners often stand in semisquatting postures (e.g., goat-gripping stance, forward attacking footwork, and pivoting footwork) during practice [8]. This may improve both the amplitude and the timing of leg muscle activation and may increase muscular strength [29].

### 4.3. Functional Balance Performance

Results revealed that the VT group participants scored 18.9% higher in the BBS than the control participants. That means that VT practitioners had better functional balance performance than the no-training controls. This result was expected and agrees with our previous study showing that long-term VT practitioners had less postural sway, especially when standing under sensory-depriving and/or conflicting environments [7]. We postulated that VT practitioners had developed better visual and vestibular senses for postural control through regular practice of the dynamic sticking-hand exercises [7]. Another possible explanation for the better functional balance performance in VT practitioners is that they had greater lower limb muscular strength than the control participants, as found in the present study. Indeed, knee muscular strength was reported to be associated with postural stability among the elderly [30].

### 4.4. Balance Self-Efficacy

Similar to tai chi practitioners [6], VT practitioners had higher ABC scores than the control participants. This may be because VT practitioners (1) had more positive experiences in performing balancing tasks (e.g., sticking-hand exercises), (2) had more chance to observe others successfully completing a balancing task, and (3) received verbal affirmation of their balance ability from others during VT training [31, 32]. However, their higher balance self-efficacy is not related to their greater lower limb muscular strength or superior functional balance performance. These findings are in contrast to Tsang and Hui-Chan [6] and Hatch et al. [33] who showed that balance confidence is correlated with knee muscular strength in older tai chi practitioners [6] and balance performance in healthy elderly people [33]. These discrepancies could be explained by the different exercise types and training volumes received by the participants, different age ranges, and the various assessment methods of muscular strength and balance performance that were used across studies. Therefore, these results cannot be compared directly.

### 4.5. Limitations and Recommendation for Further Research

Although the results were promising, there are several limitations in this study. First, a convenience sample was used and, thus, the possibility of self-selection bias cannot be excluded. For example, elders with stronger bones, muscles, balance, and confidence might be more willing to participate in VT training. Second, our sample was quite heterogeneous, including people of 40 years old or above and more than 3 months of VT experience (for the VT group). This may potentially confound the results. Third, the cross-sectional study design cannot establish causality between VT training and the physical outcomes. It would be interesting to further explore the effects of VT training on bone strength, muscular strength, body balance, and balance self-efficacy in older adults by randomized controlled trials. Finally, how VT training is associated with incidence of falls and fall-related fracture distal radius was not examined. These important clinical implications await further research.

## 5. Conclusions

Elderly VT martial arts practitioners had higher radial bone strength, greater lower limb muscular strength, better functional balance performance, and greater balance confidence than the nonpractitioners. These encouraging findings may inspire the development of VT fall-prevention exercises for the community-dwelling healthy elderly.

## Figures and Tables

**Table 1:** Participant characteristics.

	VT group	Control group	*P* value
	(*n* = 35)	(*n* = 49)	
Age (years)	62.7 ± 13.3	65.9 ± 10.5	0.219
Sex (*n*)	27 men/8 women	20 men/29 women	0.001^*^
Weight (kg)	64.3 ± 12.2	62.1 ± 11.4	0.406
Height (cm)	163.1 ± 8.1	156.8 ± 10.4	0.004^*^
Body mass index (kg/m^2^)	24.1 ± 3.7	25.2 ± 3.6	0.185
VT experience (years)	8.0 ± 9.9	0	—
Number of fall incidents in the past 6 months	0.1 ± 0.2	0.1 ± 0.5	0.381

Mean ± SD presented for continuous variables.

^*^ *P* < 0.05 for between-group comparisons.

**Table 2:** Comparison of outcome variables.

	VT group	Control group	*P* value	Effect size
	(*n* = 35)	(*n* = 49)		
Bone strength of distal radius (dominant side)				
SOS *T* score	−1.4 ± 1.4	−2.7 ± 1.6	0.035^*^	0.055
SOS *Z* score	−0.2 ± 1.3	−0.9 ± 1.0	0.009^*^	0.084

Lower limb muscular strength				
FTSTS time (s)	8.3 ± 2.2	13.9 ± 4.5	0.001^*^	0.229

Functional balance performance				
BBS score	51.5 ± 3.8	43.3 ± 7.7	0.003^*^	0.173

Balance self-efficacy				
ABC score	94.9 ± 5.6	75.9 ± 10.5	<0.001^*^	0.384

Mean ± SD presented for continuous variables.

^*^ *P* < 0.05 for between-group comparisons.

## References

[1] Gillespie L., Handoll H. (2009). Prevention of falls and fall-related injuries in older people. *Injury Prevention*.

[2] Kelsey J. L., Prill M. M., Keegan T. H. M., Tanner H. E., Bernstein A. L., Quesenberry C. P., Sidney S. (2005). Reducing the risk for distal forearm fracture: preserve bone mass, slow down, and don't fall. *Osteoporosis International*.

[3] Lachman M. E., Howland J., Tennstedt S., Jette A., Assmann S., Peterson E. W. (1998). Fear of falling and activity restriction: the survey of activities and fear of falling in the elderly (SAFE). *Journals of Gerontology B: Psychological Sciences and Social Sciences*.

[4] Binda S. M., Culham E. G., Brouwer B. (2003). Balance, muscle strength, and fear of falling in older adults. *Experimental Aging Research*.

[5] Bulat T., Hart-Hughes S., Ahmed S., Quigley P., Palacios P., Werner D. C., Foulis P. (2007). Effect of a group-based exercise program on balance in elderly. *Clinical Interventions in Aging*.

[6] Tsang W. W. N., Hui-Chan C. W. Y. (2005). Comparison of muscle torque, balance, and confindence in older Tai Chi and healthy adults. *Medicine & Science in Sports & Exercise*.

[7] Fong S. S. M., Guo X., Cheung A. P. M. (2013). Elder Chinese martial art practitioners have higher radial bone strength, hand-grip strength and better standing balance control. *ISRN Rehabilitation*.

[8] Peterson D. (2006). *Look Beyond the Pointing Finger-The Combat Philosophy of Wong Shun Leung*.

[9] Knapp K. M., Blake G. M., Spector T. D., Fogelman I. (2001). Multisite quantitative ultrasound: precision, age- and menopause-related changes, fracture discrimination, and T-score equivalence with dual-energy X-ray absorptiometry. *Osteoporosis International*.

[10] Barkmann R., Kantorovich E., Singal C., Hans D., Genant H. K., Heller M., Glüer C.-C. (2000). A new method for quantitative ultrasound measurements at multiple skeletal sites: first results of precision and fracture discrimination. *Journal of Clinical Densitometry*.

[11] Drake W. M., McClung M., Njeh C. F., Genant H. K., Rosen C., Watts N., Kendler D. L. (2001). Multisite bone ultrasound measurement on North American female reference population. *Journal of Clinical Densitometry*.

[12] BeamMed (2010). *Sunlight Miniomni Bone Sonometer User Guide*.

[13] Haney E. M., Bliziotes M. M. (2008). Male osteoporosis: new insights in an understudied disease. *Current Opinion in Rheumatology*.

[14] Ostchega Y., Harris T. B., Hirsch R., Parons V. L., Kington R., Katzoff M. (2000). Reliability and prevalence of physical performance examination assessing mobility and balance in older persons in the US: data from the third national health and nutrition examination survey. *Journal of the American Geriatrics Society*.

[15] Wallmann H. W., Evans N. S., Day C., Neelly K. R. (2013). Interrater reliability of the five-times-sit-to-stand test. *Home Health Care Management and Practice*.

[16] Ng S. S. M., Cheung S. Y., Lai L. S. W., Liu A. S. L., Ieong S. H. I., Fong S. S. M. (2013). Association of seat height and arm position on the five times sit-to-stand test times of stroke survivors. *BioMed Research International*.

[17] Berg K., Wood-Dauphinee S., Williams J. I., Gayton D. (1989). Measuring balance in the elderly: preliminary development of an instrument. *Physiotherapy Canada*.

[18] Berg K., Wood-Dauphinee S., Williams J. I. (1995). The balance scale: reliability assessment with elderly residents and patients with an acute stroke. *Scandinavian Journal of Rehabilitation Medicine*.

[19] Thorbahn L. D. B., Newton R. A. (1996). Use of the Berg balance test to predict falls in elderly persons. *Physical Therapy*.

[20] Mak M. K., Lau A. L., Law F. S., Cheung C. C., Wong I. S. (2007). Validation of the Chinese translated activities-specific balance confidence scale. *Archives of Physical Medicine and Rehabilitation*.

[21] Portney L. G., Watkins M. P. (2009). *Foundations of Clinical Research: Applications to Practice*.

[22] Yip C. (1981). *116 Wing Tsun Dummy Techniques as Demonstrated by Grandmaster Yip Man*.

[23] Ruff C., Holt B., Trinkaus E. (2006). Who's afraid of the big bad Wolff?: “Wolff's law” and bone functional adaptation. *The American Journal of Physical Anthropology*.

[24] Wolff J. (1986). *The Law of Bone Remodeling-Translated by P Maquet and R Furlong*.

[25] Madhok R., Green S. (1993). Longer term functional outcome and societal implications of upper limb fractures in the elderly. *Journal of the Royal Society of Health*.

[26] Bohannon R. W. (2006). Reference values for the five-repetition sit-to-stand test: a descriptive meta-analysis of data from elders. *Perceptual and Motor Skills*.

[27] O'Donovan O., Cheung J., Catley M., McGregor A. H., Strutton P. H. (2006). An investigation of leg and trunk strength and reaction times of hard-style martial arts practitioners. *Journal of Sports Science and Medicine*.

[28] Probst M. M., Fletcher R., Seelig D. S. (2007). A comparison of lower-body flexibility, strength, and knee stability between karate athletes and active controls. *Journal of Strength and Conditioning Research*.

[29] Rutherford O. M., Jones D. A. (1986). The role of learning and coordination in strength training. *European Journal of Applied Physiology and Occupational Physiology*.

[30] Wu G., Zhao F., Zhou X., Wei L. (2002). Improvement of isokinetic knee extensor strength and reduction of postural sway in the elderly from long-term Tai Chi exercise. *Archives of Physical Medicine and Rehabilitation*.

[31] Bandura A., Bandura A. (1995). Exercise of personal and collective efficacy in changing societies. *Self-Efficacy in Changing Societies*.

[32] Bandura A. (1977). Self-efficacy: toward a unifying theory of behavioral change. *Psychological Review*.

[33] Hatch J., Gill-Body K. M., Portney L. G. (2003). Determinants of balance confidence in community-dwelling elderly people. *Physical Therapy*.

